# Supplementary material for: Spatial patterns and determinants of low utilization of delivery care service and postnatal check-up within 2 months following birth in Ethiopia: Bivariate analysis
Source: PLoS One. 2024 Jan 24;19(1):e0297038. doi: 10.1371/journal.pone.0297038 (PMC10807769; doi:10.1371/journal.pone.0297038)
Supplement: Attachment — Submitted filename: Peer_Review final.docx [file pone.0297038.s001.docx]

**Date:** Monday September 18th, 2023

**Journal:** PLOS ONE

Dear Editor-in-Chief, Associate Editor, and Authors

Thank you for the opportunity to review the manuscript entitled " Spatial Distribution of Home Delivery and Baby not Receive Postnatal Check-up within 2 Months after Birth in Ethiopia: Bivariate Analysis".

- **Summary:**

In this study, the authors conducted a comprehensive analysis using bivariate binary logistic regression to investigate the spatial patterns and determining factors associated with the low utilization of delivery care (DC) services and postnatal check-ups (PNC) following live births in Ethiopia. This research sets itself apart from existing literature by delving into the relationship between home deliveries and PNC utilization while considering their geographical distribution.

To gather data, the authors utilized information from the 2016 Ethiopian Demographic and Health Survey, ensuring a robust sample size for their analysis to draw a reliable conclusion. The significance of this study lies in its contribution to our understanding of the factors influencing maternal and infant health. It offers valuable insights that can aid healthcare providers and policymakers in formulating future plans and strategies to enhance postnatal services.

In conclusion, the authors have stated a multitude of diverse socioeconomic, demographic, and geographical factors that significantly influence the utilization of delivery and postnatal care services. These findings have been effectively visualized through informative graphics and tables. This study holds considerable clinical and research significance; nevertheless, there are certain comments that warrant further discussion and response.

- **Comments to Authors:**

**Title:**

- The title of the study is relatively good. However, the authors should revise the title to be more inclusive and precise to the study’s objectives and aims (such as the determinants of PNC utilization).

**Introduction:**

- It is clear, concise, and straightforward, and the rationale and aim of the study are well-stated. In addition, important definitions were illustrated clearly.

**Methods:**

- The authors conducted a database study and obtained ethical approval as requested.
- Sampling method was illustrated properly.

**Results:**

- The authors conducted a thorough analysis of the results, supported by sufficient amount of data justifying the conclusion. Nevertheless, the analysis assumed a linear relationship between dependent and independent variables, overlooking potential confounding factors that could impact the accuracy of correlations. Therefore, further research may be warranted to validate these hypotheses.

**Discussion:**

- In the discussion section, the authors have effectively compared their findings with those of similar studies and offered reasonable explanations for both the similarities and differences. It is crucial, however, to avoid over interpreting the data.
- While the authors did highlight the clinical implications of their study's findings, it is worth noting that they missed addressing the study's limitations. It is advisable to have a transparent discussion of these limitations to provide a more comprehensive understanding of the research's scope and potential constraint.
- Further clinical application should be mentioned for policy makers, together with future direction and recommendation.

**Conclusion:**

- Correctly answered the research question.
- Authors should consider conciseness of conclusion to represent the whole findings.

**Minor:**

- English editing and proofreading by a native English speaker needed. The authors should correct some typos and language mistakes.
