# Supplementary material for: Spatial patterns and determinants of low utilization of delivery care service and postnatal check-up within 2 months following birth in Ethiopia: Bivariate analysis
Source: PLoS One. 2024 Jan 24;19(1):e0297038. doi: 10.1371/journal.pone.0297038 (PMC10807769; doi:10.1371/journal.pone.0297038)
Supplement: Attachment — Submitted filename: Author response.docx [file pone.0297038.s002.docx]

**Author's Response to Reviewers' and Academic Editors' Comments:**

Manuscript number: PONE-D-23-23873

Title: Spatial Distribution of Home Delivery and Baby not Receive Postnatal Check-up within 2 Months after Birth in Ethiopia: Bivariate Analysis

We are thankful to the academic editor's and reviewers for the thoroughly review of our manuscript, encouraging remarks, their valuable comments and gave us the chance to resubmit the revised version. Following are replays that have been addressed in response to the academic editor's and reviewers' comments:

**Academic Editors' Comments:**

Comment:

1. When submitting your revision, we need you to address these additional requirements.

Please ensure that your manuscript meets PLOS ONE's style requirements, including those for file naming. The PLOS ONE style templates can be found at 
<https://journals.plos.org/plosone/s/file?id=wjVg/PLOSOne_formatting_sample_main_body.pdf> and 
<https://journals.plos.org/plosone/s/file?id=ba62/PLOSOne_formatting_sample_title_authors_affiliations.pdf>

Response:

In order to comply with the journal's standards, the manuscript was thoroughly revised using the PLOS ONE’s style template, which can be seen in the links provided above (please see the marked-up and unmarked copies of the revised manuscript).

Comment:

2. We note that you have indicated that data from this study are available upon request. PLOS only allows data to be available upon request if there are legal or ethical restrictions on sharing data publicly. For more information on unacceptable data access restrictions, please see <http://journals.plos.org/plosone/s/data-availability#loc-unacceptable-data-access-restrictions>.

In your revised cover letter, please address the following prompts:

a) If there are ethical or legal restrictions on sharing a de-identified data set, please explain them in detail (e.g., data contain potentially sensitive information, data are owned by a third-party organization, etc.) and who has imposed them (e.g., an ethics committee). Please also provide contact information for a data access committee, ethics committee, or other institutional body to which data requests may be sent.

b) If there are no restrictions, please upload the minimal anonymized data set necessary to replicate your study findings as either Supporting Information files or to a stable, public repository and provide us with the relevant URLs, DOIs, or accession numbers. For a list of acceptable repositories, please see <http://journals.plos.org/plosone/s/data-availability#loc-recommended-repositories>.

We will update your Data Availability statement on your behalf to reflect the information you provide.

Response:

Dear Editor, thank you for your feedback and guidance. We apologize for the confusion regarding the availability of our data. Upon reviewing the PLOS ONE data availability policy, we understand that PLOS only allows data to be available upon request if there are legal or ethical restrictions on sharing data publicly. We have updated the data availability statement accordingly and mentioned the changes in the revised cover letter

There is legal restrictions on sharing a de-identified data set as it is owned by a third-party (Demographic and health survey program). A written permission was obtained from the major demographic and health survey (DHS) program after online request to the program via the link [www.dhsprogram.com](http://www.dhsprogram.com). The permission letter was attached as file type “other” in the previous submission. Any interested body can register and access the data through the link provided above.

Comment:

3. We note that you have stated that you will provide repository information for your data at acceptance. Should your manuscript be accepted for publication, we will hold it until you provide the relevant accession numbers or DOIs necessary to access your data. If you wish to make changes to your Data Availability statement, please describe these changes in your cover letter and we will update your Data Availability statement to reflect the information you provide.

Response:

Data from the major demographic and health survey program are accessible due to restrictions imposed by third parties. The institute can be contacted by interested researchers through online registration via the link [www.dhsprogram.com](http://www.dhsprogram.com) to request access to the data. Following the editor's suggestions, the manuscript's data availability section has been revised and it was mentioned in the revised cover letter.

Comment:

4. We note that Figures 2, 3 and 4 in your submission contain map images which may be copyrighted. All PLOS content is published under the Creative Commons Attribution License (CC BY 4.0), which means that the manuscript, images, and Supporting Information files will be freely available online, and any third party is permitted to access, download, copy, distribute, and use these materials in any way, even commercially, with proper attribution. For these reasons, we cannot publish previously copyrighted maps or satellite images created using proprietary data, such as Google software (Google Maps, Street View, and Earth). For more information, see our copyright guidelines: <http://journals.plos.org/plosone/s/licenses-and-copyright>.
 
We require you to either present written permission from the copyright holder to publish these figures specifically under the CC BY 4.0 license, or (2) remove the figures from your submission:
 
a. you may seek permission from the original copyright holder of Figures 2, 3 and 4 to publish the content specifically under the CC BY 4.0 license.  
 
We recommend that you contact the original copyright holder with the Content Permission Form (<http://journals.plos.org/plosone/s/file?id=7c09/content-permission-form.pdf>) and the following text:
“I request permission for the open-access journal PLOS ONE to publish XXX under the Creative Commons Attribution License (CCAL) CC BY 4.0 (<http://creativecommons.org/licenses/by/4.0/>). Please be aware that this license allows unrestricted use and distribution, even commercially, by third parties. Please reply and provide explicit written permission to publish XXX under a CC BY license and complete the attached form.”
 
Please upload the completed Content Permission Form or other proof of granted permissions as an "Other" file with your submission.
 
In the figure caption of the copyrighted figure, please include the following text: “Reprinted from [ref] under a CC BY license, with permission from [name of publisher], original copyright [original copyright year].”
 
b. If you are unable to obtain permission from the original copyright holder to publish these figures under the CC BY 4.0 license or if the copyright holder’s requirements are incompatible with the CC BY 4.0 license, please either i) remove the figure or ii) supply a replacement figure that complies with the CC BY 4.0 license. Please check copyright information on all replacement figures and update the figure caption with source information. If applicable, please specify in the figure caption text when a figure is similar but not identical to the original image and is therefore for illustrative purposes only.
The following resources for replacing copyrighted map figures may be helpful:
 
USGS National Map Viewer (public domain): <http://viewer.nationalmap.gov/viewer/>
The Gateway to Astronaut Photography of Earth (public domain): <http://eol.jsc.nasa.gov/sseop/clickmap/>
Maps at the CIA (public domain): <https://www.cia.gov/library/publications/the-world-factbook/index.html> and <https://www.cia.gov/library/publications/cia-maps-publications/index.html>
NASA Earth Observatory (public domain): <http://earthobservatory.nasa.gov/>
Landsat: <http://landsat.visibleearth.nasa.gov/>
USGS EROS (Earth Resources Observatory and Science (EROS) Center) (public domain): [http://eros.usgs.gov/#](http://eros.usgs.gov/)
Natural Earth (public domain): <http://www.naturalearthdata.com/>

Response:

It is legitimate and crucial to address the problem of map copyright. Before utilizing it, we were aware of that. The maps depict the official map of Ethiopia and utilized for the purpose of illustrating some findings of the study. The maps were not copyrighted directly from third parties, rather drown by the current author using Arc Map version 10.8 software. The shape files we used to create the maps belonged to the Ethiopian Central Statistical Agency (CSA), later named as Ethiopian statistical service (ESS). We are thankful to the Ethiopian statistical service (ESS) for providing the shape files.

**Reviewers' comments:**

Reviewer's Responses to Questions

Comments to the Author

1. Is the manuscript technically sound, and do the data support the conclusions?

The manuscript must describe a technically sound piece of scientific research with data that supports the conclusions. Experiments must have been conducted rigorously, with appropriate controls, replication, and sample sizes. The conclusions must be drawn appropriately based on the data presented.

Reviewer #1: Yes

Reviewer #2: Yes

Response: It is okay, thank you.

2. Has the statistical analysis been performed appropriately and rigorously?

Reviewer #1: Yes

Reviewer #2: I Don't Know

Response:

We would like to aware the second reviewer that the statistical analysis has been performed appropriately and rigorously.

3. Have the authors made all data underlying the findings in their manuscript fully available?

The [PLOS Data policy](http://www.plosone.org/static/policies.action#sharing) requires authors to make all data underlying the findings described in their manuscript fully available without restriction, with rare exception (please refer to the Data Availability Statement in the manuscript PDF file). The data should be provided as part of the manuscript or its supporting information, or deposited to a public repository. For example, in addition to summary statistics, the data points behind means, medians and variance measures should be available. If there are restrictions on publicly sharing data—e.g. participant privacy or use of data from a third party—those must be specified.

Reviewer #1: Yes

Reviewer #2: Yes

Response: It is okay, thank you.

4. Is the manuscript presented in an intelligible fashion and written in standard English?

PLOS ONE does not copyedit accepted manuscripts, so the language in submitted articles must be clear, correct, and unambiguous. Any typographical or grammatical errors should be corrected at revision, so please note any specific errors here.

Reviewer #1: Yes

Reviewer #2: No

Response:

We have taken a step to correct typographical or grammatical errors that need to be corrected (see the marked-up copy of the manuscript in the revised submission).

5. Review Comments to the Author

Please use the space provided to explain your answers to the questions above. You may also include additional comments for the author, including concerns about dual publication, research ethics, or publication ethics. (Please upload your review as an attachment if it exceeds 20,000 characters)

**Reviewer #1:**

A good effort's to highlight this topic by providing with detailed statistical analysis, but there are many already provided literatures which focuses on postnatal checkup and delivery care. This study cannot add much new information to the existing literature.

Response:

It is important for research studies to provide new insights or contribute novel findings to the field. Even though there are already numerous published studies that have extensively covered the topic of postnatal checkups and delivery care, the current manuscript should be assessed for its potential to add new information, perspectives, or approaches to the existing literatures. By this study it is tried to present a detailed statistical analysis, such as spatial and bivariate analysis of the two response variables (Delivery care and postnatal check-ups), thus it is essential to evaluate its potential contribution.

**Reviewer #2:  Comments to Authors:**
Title:
• The title of the study is relatively good. However, the authors should revise the title to be more inclusive and precise to the study’s objectives and aims (such as the determinants of PNC utilization).

Response:

Dear reviewer, thank you for your guidance on revising the title of our study. We appreciate your suggestion and have made the necessary changes. The revised title now reads: “Spatial patterns and determinants of low utilization of delivery care service and postnatal check-up within 2 months following birth in Ethiopia: Bivariate analysis” (see the marked-up copy of the revised submission)
Introduction:
• It is clear, concise, and straightforward, and the rationale and aim of the study are well-stated. In addition, important definitions were illustrated clearly.

Response: It is okay, thank you.
Methods:
• The authors conducted a database study and obtained ethical approval as requested.
• Sampling method was illustrated properly.

Response: It is okay, thank you.

Results:
• The authors conducted a thorough analysis of the results, supported by sufficient amount of data justifying the conclusion. Nevertheless, the analysis assumed a linear relationship between dependent and independent variables, overlooking potential confounding factors that could impact the accuracy of correlations. Therefore, further research may be warranted to validate these hypotheses.

Response:

Dear reviewer, thank you for your feedback on our analysis. We appreciate your point about potential confounding factors that could impact the accuracy of correlations and agree that further research may be warranted to validate our hypotheses. In future studies, we will take into consideration potential confounding factors and explore non-linear relationships between dependent and independent variables. This will help us provide a more comprehensive understanding of the determinants of delivery and postnatal care utilization.

Discussion:
• In the discussion section, the authors have effectively compared their findings with those of similar studies and offered reasonable explanations for both the similarities and differences. It is crucial, however, to avoid over interpreting the data.

Response:

Thank you for pointing out this potential issue and we appreciate your guidance in maintaining the integrity of our study. We understand the importance of not over-interpreting the data and have taken steps to avoid this in our revised submission (see the marked-up copy of the revised submission).
• While the authors did highlight the clinical implications of their study's findings, it is worth noting that they missed addressing the study's limitations. It is advisable to have a transparent discussion of these limitations to provide a more comprehensive understanding of the research's scope and potential constraint.

Response:

Thank you for your feedback. We have included a section in the revised submission dedicated to discussing the limitations of our study. This section provides an overview of potential constraints and acknowledges any biases or confounding factors that may have influenced our results. Additionally, we have included suggestions for future research to address these limitations and further explore the topic. These recommendations aim to guide other researchers in designing studies that can build upon our findings and contribute to the field (see the marked-up copy in the revised submission).
• Further clinical application should be mentioned for policy makers, together with future direction and recommendation.
Response:

Thank you for your suggestion. We have added a section in the revised submission that discusses the potential clinical applications of our findings and provides recommendations for policy makers. This section highlights the practical implications of our study and how the results can be used to inform decision-making and policy development in the relevant field.

Furthermore, we have included a subsection on future directions and recommendations, which outlines specific areas for further research and investigation. These recommendations are intended to guide future studies and help expand our understanding of the topic (see the marked-up copy of the revised submission).
Conclusion:
• Correctly answered the research question.

Response: thank you.
• Authors should consider conciseness of conclusion to represent the whole findings.

Response:

Thank you for your suggestion. We have revised the conclusion to ensure it represents the entirety of our findings in a concise manner. We have carefully summarized the key results and implications of our study, providing a clear and succinct conclusion that captures the main takeaways for both researchers and practitioners in the field (see the marked-up copy).

Minor:
• English editing and proofreading by a native English speaker needed. The authors should correct some typos and language mistakes.

Response:

We have taken the step of having the revised manuscript professionally edited and proofread for English language errors. However, we may still have some typos and language mistakes as none of the authors in this study are native English speakers. Thank you for your guidance and we apologize for any typos and language mistakes.

Thank you dear editors and reviewers for your guidance and comments!

Sincerely yours,

Shegaw Mamaru Awoke
